# Mechanisms of circular RNAs in diabetic cardiomyopathy: biological characteristics and clinical prospects

**DOI:** 10.3389/fgene.2025.1665571

**Published:** 2025-09-24

**Authors:** Yunfei Guan, Quancheng Han, Meng Wang, Jianguo Xu, Xiujuan Liu

**Affiliations:** ^1^ Ningjin County Traditional Chinese Medicine Hospital, Dezhou, China; ^2^ Shandong University of Traditional Chinese Medicine, Jinan, China; ^3^ Shandong Academy of Chinese Medicine, Jinan, China; ^4^ Affiliated Hospital of Shandong University of Traditional Chinese Medicine, Jinan, China

**Keywords:** circRNA, diabetic cardiomyopathy, metabolic dysregulation, oxidativestress, programmed cell death, clinical application

## Abstract

Diabetic cardiomyopathy (DCM) is a specific form of heart disease induced by diabetes, characterized by myocardial fibrosis, oxidative stress, metabolic dysregulation, and cardiomyocyte death. In recent years, circular RNAs (circRNAs), a novel class of non-coding RNAs, have gained increasing attention due to their unique covalently closed structure, high stability, and critical regulatory roles in various diseases. While extensive studies have been conducted on microRNAs (miRNAs) and long non-coding RNAs (lncRNAs) in the context of DCM, research on circRNAs remains relatively limited and fragmented. Existing reviews often focus on specific aspects without providing a systematic and comprehensive overview. This review aims to summarize the current progress in circRNA research related to DCM, with a particular focus on the molecular mechanisms and regulatory networks through which circRNAs influence metabolic disorders, oxidative stress, myocardial fibrosis, and programmed cell death. In addition, the potential of circRNAs as diagnostic biomarkers and therapeutic targets is evaluated, along with an in-depth discussion of current challenges and future research directions. This work is intended to offer theoretical insights and reference value for both fundamental and translational studies of circRNAs in DCM.

## 1 Introduction

Diabetic cardiomyopathy (DCM) is a common cardiovascular complication in patients with diabetes mellitus (DM), characterized by structural and functional abnormalities of the heart in the absence of coronary artery disease or hypertension (Giraldo-Gonzalez et al., 2025; Weeks et al., 2025; Yu et al., 2025). The pathological features of DCM primarily include myocardial fibrosis, oxidative stress, metabolic dysregulation, and pyroptosis (Guo et al., 2025; Shang et al., 2025; Sun J. et al., 2025; Zhang et al., 2025). With the global prevalence of DM continuing to rise, DCM has emerged as one of the major causes of heart failure and cardiovascular mortality (Grobman et al., 2025; Nagori et al., 2025). However, the underlying mechanisms of DCM remain incompletely understood, and current clinical treatments are still limited (Armillotta et al., 2025). Therefore, it is imperative to explore novel molecular targets and therapeutic strategies.

Circular RNAs (circRNAs), a class of non-coding RNAs with covalently closed loop structures, have attracted increasing attention in recent years due to their remarkable stability, tissue-specific expression patterns, and evolutionary conservation (Kang et al., 2025; Yang Y. et al., 2025). CircRNAs are primarily generated through back-splicing events and are tightly regulated by RNA-binding proteins (RBPs) (Wang et al., 2025a). Emerging studies have shown that circRNAs can participate in a variety of biological processes, such as metabolism, apoptosis, and fibrosis, by acting as microRNA (miRNA) sponges, modulating gene transcription, or interacting with proteins (Boichenko et al., 2025; Gupta et al., 2025; Kim and Ryu, 2025; Liao et al., 2025). These findings suggest that circRNAs play critical roles in cardiovascular diseases. Nevertheless, the specific regulatory mechanisms of circRNAs in DCM, as well as their translational potential, remain largely unexplored.

Although some studies have identified individual circRNAs—such as circRNA_000203 and circHIPK3—as key regulators in diabetic myocardial fibrosis, oxidative stress, and cardiomyocyte injury, a comprehensive understanding of the circRNA-mediated regulatory network, molecular mechanisms, and clinical relevance in DCM is still lacking (Liao et al., 2025; Li et al., 2020; Wang Y. et al., 2024; Xu et al., 2020). Therefore, a systematic investigation into the biological functions of circRNAs in DCM, along with an exploration of their potential as novel biomarkers or therapeutic targets, is of great importance for early diagnosis and precision treatment of this condition.

This review begins with a discussion of the biological characteristics of circRNAs, summarizes their roles in the pathogenesis of DCM, and highlights their potential clinical applications, aiming to provide new insights for the prevention and management of DCM.

## 2 Biological regulatory mechanisms of circRNAs

### 2.1 Structure and characteristics of circRNAs

CircRNAs are a class of non-coding RNA molecules characterized by covalently closed circular structures, which distinguish them from traditional linear RNAs (Fang et al., 2025). Unlike linear RNAs, circRNAs lack both a 5′ cap and a 3′ poly(A) tail, making them resistant to exonuclease-mediated degradation and thereby conferring high stability within the cellular environment (Ding et al., 2025; Greco et al., 2025). This stability allows circRNAs to persist over extended periods in complex intracellular contexts and to participate in the regulation of various biological processes (Sanati and Ghafouri-Fard, 2025). The discovery of circRNAs has significantly expanded our understanding of the RNA world and established a new and important subfield within non-coding RNA research.

CircRNAs exhibit structural diversity and are mainly classified into four types: exonic circRNAs (ecircRNAs), circular intronic RNAs (ciRNAs), exon-intron circRNAs (EIciRNAs), and tRNA-derived circRNAs (tricRNAs) (Yang Y. et al., 2025; Lv et al., 2025; Schmidt and Matera, 2020). Among them, ecircRNAs are the most abundant and are formed by back-splicing of one or more exons (Lin et al., 2021). They are predominantly located in the cytoplasm, where they act as competing endogenous RNAs (ceRNAs) by sponging miRNAs or interacting with proteins to regulate gene expression (Xi et al., 2025). ciRNAs, derived from retained intronic sequences, are typically localized in the nucleus and regulate host gene transcription by interacting with transcriptional machinery such as RNA polymerase II (Shafaghat et al., 2025). EIciRNAs contain both exonic and intronic regions and also tend to function in the nucleus, for example by recruiting U1 small nuclear ribonucleoproteins (snRNPs) to enhance the transcription of host genes (Li et al., 2021). TricRNAs represent a relatively new subclass of circRNAs, generated from tRNA precursors under stress conditions, and are thought to be involved in cellular stress responses, although their precise functions remain to be fully elucidated (Robic and Kühn, 2020).

The biogenesis of circRNAs mainly relies on a back-splicing mechanism, which differs from canonical linear RNA splicing (Hatzimanolis et al., 2025). In back-splicing, a downstream 5′ splice donor site is covalently linked to an upstream 3′ splice acceptor site, resulting in the formation of a closed circular RNA molecule (Sur et al., 2025). This process is regulated by multiple factors, including genomic structural features, RBPs, and cis-acting elements (Jiang et al., 2025). For instance, reverse complementary sequences within flanking introns—such as Alu repeats—can facilitate circularization *via* base pairing. Meanwhile, RBPs such as QKI, MBL, and FUS can bind specific RNA motifs and promote the circularization process (Srinivasan et al., 2025; Wang et al., 2025b; Zhu et al., 2025). Additionally, components of the spliceosome and their cofactors, including SF3B1 and U2AF65, also participate in circRNA formation, underscoring the highly regulated nature of circRNA biogenesis (Hollander et al., 2016; Paira and Borden, 2025).

In terms of intracellular localization, circRNAs exhibit clear subcellular distribution preferences. EcircRNAs are primarily localized in the cytoplasm, where they regulate post-transcriptional gene expression by interacting with miRNAs or proteins (Liu et al., 2025). A well-known example is CDR1as (ciRS-7), which acts as a sponge for miR-7 to modulate the expression of its target genes, thereby influencing cellular proliferation and differentiation (Lou et al., 2024). In contrast, ciRNAs and EIciRNAs are enriched in the nucleus, where they modulate transcriptional activity by interacting with transcription complexes or chromatin-modifying factors (Song W. et al., 2024). These distinct localization patterns reflect the functional diversity of circRNAs and suggest that they may exert context-dependent biological effects in different cellular compartments.

CircRNA expression is highly tissue-specific and developmentally regulated. Distinct circRNA expression profiles have been observed across various tissues and cell types, with some circRNAs being specifically upregulated or downregulated under particular pathological conditions, such as cancer, cardiovascular disease, or neurodegenerative disorders (Ali et al., 2025; Mohammadpour et al., 2025; Sun M. et al., 2025). This context-dependent expression pattern makes circRNAs attractive candidates for disease biomarkers or therapeutic targets. In the case of DCM, certain circRNAs have been found to regulate key pathological processes such as myocardial fibrosis, oxidative stress, and metabolic dysregulation, offering new directions for clinical diagnosis and treatment (Rai et al., 2020).

### 2.2 Biological functions of circRNAs

#### 2.2.1 miRNA sponging effect (core mechanism)

The covalently closed circular structure of circRNAs endows them with a unique capacity to act as miRNA sponges (Tarhriz et al., 2025). Unlike linear RNAs, circRNAs lack free 5′ and 3′ ends, rendering them resistant to exonuclease degradation and allowing them to exhibit a prolonged intracellular half-life, often exceeding 48 h (Gao et al., 2025). This stability enables circRNAs to continuously function as miRNA sponges. Structurally, efficient miRNA sponge circRNAs typically harbor multiple miRNA response elements (MREs), which are 7–8 nucleotide conserved sequences complementary to the “seed region” of specific miRNAs (Li L. et al., 2025). For example, the well-known ciRS-7 contains more than 70 highly conserved binding sites for miR-7, each forming canonical Watson–Crick base pairing with miR-7 (Li and Wang, 2024). Notably, the circular conformation of circRNAs may promote multivalent binding, whereby a single circRNA molecule can simultaneously bind several miRNA molecules, forming complex RNA–protein interaction networks (Chen M. et al., 2025).

The miRNA sponging effect of circRNAs exhibits notable spatiotemporal specificity (Ghafouri-Fard et al., 2022). During development, some circRNAs show stage-specific expression dynamics that are inversely correlated with the expression of key miRNAs (Ghafouri-Fard et al., 2022). In terms of subcellular localization, most circRNAs with miRNA sponge activity are enriched in the cytoplasm, colocalizing with their target miRNAs and mRNAs (Galli et al., 2025). Recent studies have demonstrated that certain circRNAs can form biomolecular condensates *via* liquid–liquid phase separation, enhancing the local efficiency of miRNA sequestration (Guo et al., 2024). This dynamic regulation enables circRNAs to respond to cellular state changes and selectively activate sponging activity under specific temporal and spatial conditions (Ku et al., 2025).

Under pathological conditions, the circRNA–miRNA axis serves as a critical component of gene expression regulation and is extensively involved in the onset and progression of various diseases. Studies have demonstrated that circHIPK3 is markedly upregulated under multiple stress conditions and acts as a sponge for miR-124, thereby activating signaling pathways such as STAT3 and PI3K/Akt, which promote cell proliferation and inhibit apoptosis (Feng et al., 2022). This pathological role has been confirmed in both cancer and cardiovascular diseases. Similarly, circZNF609 modulates cardiomyocyte apoptosis during ischemia-reperfusion injury by regulating miR-214, thus participating in myocardial repair processes (Wang S. et al., 2022). CircFoxo3, highly expressed in aged cardiomyocytes, interacts with miR-138 or miR-433 to regulate oxidative stress-related factors and pro-apoptotic proteins, thereby promoting cardiomyocyte senescence and dysfunction (Zhao M. et al., 2025). These findings highlight that, in various pathological contexts, circRNAs function as ceRNAs to fine-tune cell fate decisions. By orchestrating inflammation, fibrosis, and metabolic dysregulation through tissue-specific and pathway-selective mechanisms, circRNAs represent promising therapeutic targets.

Beyond the classical competitive binding mechanism, circRNA-mediated miRNA regulation encompasses multilayered modes of action. Certain circRNAs, such as circCCDC66, enhance regulatory specificity by forming ternary complexes with miRNAs and mRNAs (Wang X. et al., 2022; Wang and Fu, 2025). Others, like circZNF91, undergo conformational changes upon miRNA binding, exposing hidden protein-binding domains and recruiting additional effectors (Zeng et al., 2021). This adaptive regulation significantly expands the dynamic range of the circRNA–miRNA interaction network.

#### 2.2.2 Protein interactions

The interaction between circRNAs and proteins is grounded in the unique structural properties of circRNAs. Their covalently closed circular structure creates distinct three-dimensional conformations, exposing protein-binding interfaces that differ from those of linear RNAs (Amelimojarad and Amelimojarad, 2025). Studies have identified specific protein-binding domains (PBDs) on circRNAs that engage proteins through hydrogen bonding, van der Waals forces, and electrostatic interactions (Amelimojarad and Amelimojarad, 2025). Some circRNAs, such as circFoxo3, depend on internally formed G-quadruplex structures for high-affinity protein binding (Joshi et al., 2025). Importantly, the circular conformation enhances the stability of circRNA–protein complexes, giving them longer binding half-lives than linear RNA–protein interactions—an advantage attributed to the absence of exonuclease-sensitive termini (Wang et al., 2025c).

circRNA–protein interactions form highly dynamic regulatory networks. At different stages of the cell cycle, circRNAs such as circLIMK1-005 and circ-Foxo3 exhibit cyclic changes in their binding with specific cyclins (e.g., CDK2, cyclin D1) (Du et al., 2016; Yang X. et al., 2025). This dynamic nature also manifests in subcellular localization: nuclear circRNAs (e.g., CircRNA ITCH) interact with transcriptional machinery to regulate gene expression, whereas cytoplasmic circRNAs (e.g., circPABPN1) primarily influence translational processes (Abdelmohsen et al., 2017; Liu et al., 2022). circRNA–protein interactions exhibit diverse functional mechanisms. Acting as protein “sponges,” circAmotl1 binds PDK1 and AKT1, regulating their subcellular localization and activity (Zeng et al., 2017). As molecular “scaffolds,” circACC1 simultaneously binds the β and γ subunits of AMPK to facilitate the formation of the active enzyme complex (Li et al., 2019). Some circRNAs, such as circMBL, function as molecular “allosteric modulators,” inducing conformational changes in their binding partners that influence their interactions with other RNAs (Pamudurti et al., 2022). These multifaceted mechanisms underscore the central role of circRNAs in regulating intracellular signal transduction.

#### 2.2.3 Translation into functional peptides

The translational potential of circRNAs arises from internal translation initiation elements embedded within their circular structure (Lin et al., 2025). Unlike linear mRNAs, circRNAs do not rely on a 5′ cap for translation initiation. Instead, they are translated *via* internal ribosome entry sites (IRES) or m6A-mediated mechanisms (Lin et al., 2025). Studies have shown that translatable circRNAs often contain complete ORFs, with start codons located 50–200 nucleotides downstream of IRES elements (Madern et al., 2025). Certain circRNAs (e.g., circZNF609) initiate translation independently of m6A methyltransferase METTL3 or RNA sequence elements such as IRES, but rely on specific RBPs to form an assembly platform for the translation initiation complex (Ho-Xuan et al., 2020). Notably, m6A modifications can recruit initiation factors such as YTHDF3 and eIF4G2 to initiate circRNA translation even in the absence of IRES elements, a mechanism particularly active under stress conditions such as hypoxia (Sun X. et al., 2025; Wang Z. W. et al., 2025).

The peptides translated from circRNAs are structurally and functionally unique. Due to ORF length constraints (typically <300 amino acids), these peptides often lack full protein domains but retain key functional motifs (Deng et al., 2024). The 185-aa peptide encoded by circFBXW7 contains a complete functional domain, enabling it to competitively interact with USP28 and thereby “release” FBXW7α to degrade c-Myc, ultimately regulating cell cycle progression (Yang et al., 2018). Interestingly, certain circRNAs (e.g., circPPP1R12A) can generate alternative peptide products distinct from their parental genes, thereby expanding the coding potential of the genome (Mookherjee et al., 2022). Although mass spectrometry analyses indicate that circRNA-derived peptides typically exist at low intracellular concentrations (nM to pM range), their specific localization and interaction profiles allow them to exert significant biological effects (Meng et al., 2022).

The translation of circRNAs is tightly regulated at multiple levels. At the transcriptional level, the composition of exons resulting from back-splicing determines ORF integrity (Harsij et al., 2025). Post-transcriptional modifications, particularly dynamic m6A methylation and demethylation, act as molecular switches controlling translation (Xu et al., 2025). Environmental stresses such as oxidative stress can significantly enhance the translational efficiency of certain circRNAs (e.g., circSHPRH), often involving reorganization of stress granules (Sun M. et al., 2025). Recent studies also suggest that some circRNA-derived peptides, such as those encoded by circβ-catenin, can negatively regulate their own translation, creating autoregulatory feedback loops. These multilayered regulatory mechanisms ensure that circRNA-derived peptides are expressed under precise spatiotemporal conditions (SHI et al., 2022).

**FIGURE 1:**
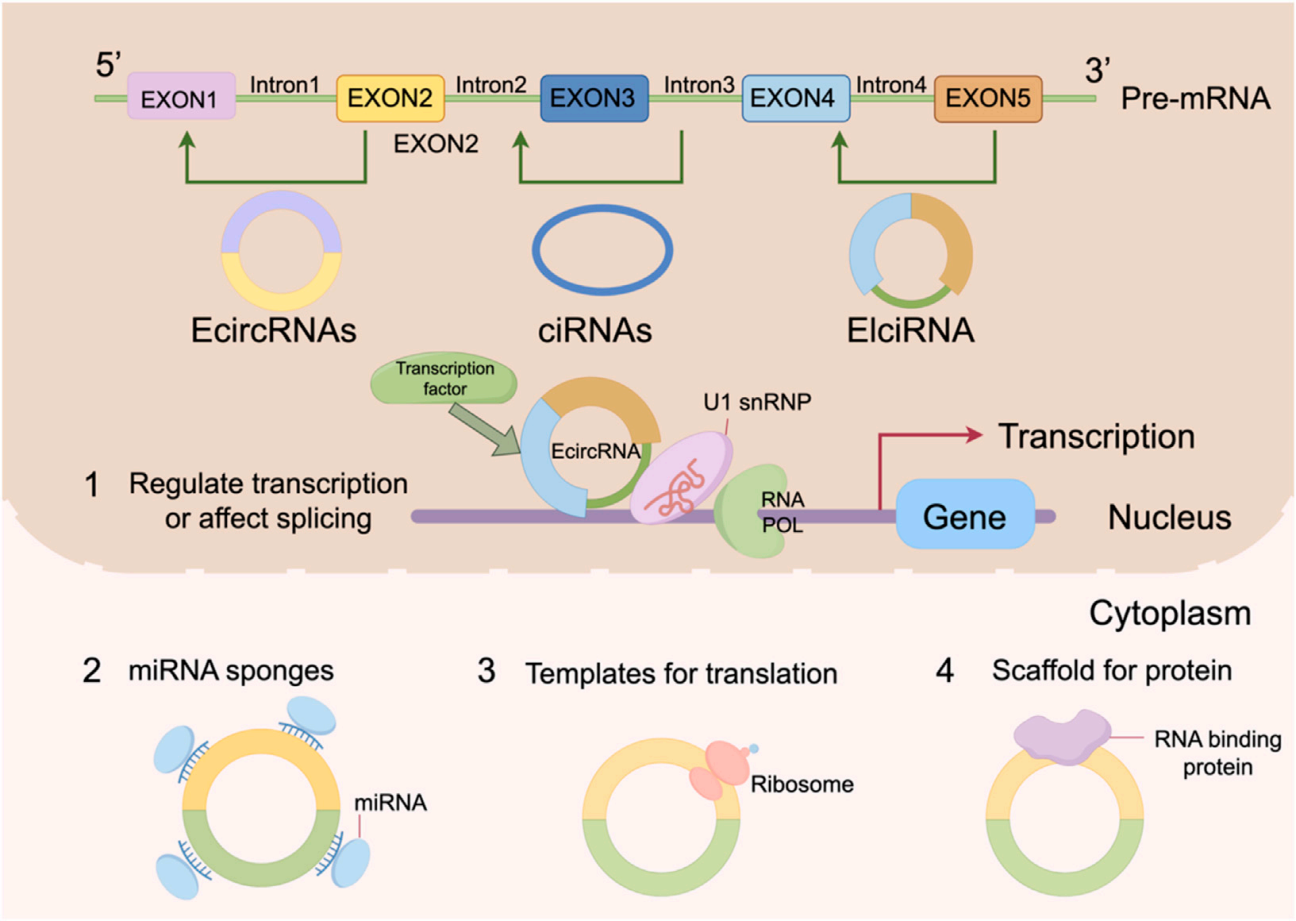
The regulatory mechanism of circRNAs.

The regulatory mechanism of circRNAs is shown in Figure 1.

### 2.3 CircRNA metabolism and degradation

The degradation of circRNAs *in vivo* is predominantly characterized by “endonuclease-mediated” pathways. Due to the absence of 5′ and 3′ termini, exonucleases generally cannot act directly, making circRNA turnover dependent on multiple intracellular endonucleolytic mechanisms. Recent studies employing *in vivo* and *ex vivo* models have further demonstrated that circRNAs are not “nondegradable,” but instead exist in a dynamic balance between homeostasis and stress responses: in aged brain tissues, abundant circRNAs can accumulate over the long term, yet their half-lives remain finely regulated by specific ribonucleases and epitranscriptomic marks such as m6A (Xu F. et al., 2024). Among these, m6A-dependent degradation represents the most prominent selective pathway under homeostatic conditions. The m6A reader protein YTH N6-methyladenosine RNA binding protein 2 (YTHDF2) recognizes methylated circRNAs and recruits RNase P/MRP endoribonuclease complex *via* the adaptor protein heat-responsive protein 12 (HRSP12) to facilitate cleavage; conversely, demethylases such as alkB homolog 5 (ALKBH5) and fat mass and obesity-associated protein (FTO) are thought to attenuate this degradation process. Multiple systematic reviews and experimental studies published between 2024 and 2025 have consistently identified this axis as a critical regulatory hub in the interplay between m6A modification and circRNA stability (Liu F. et al., 2024).

miRNA/Argonaute 2 (AGO2)-mediated “slicing-type” degradation highlights the decisive role of sequence complementarity. When circRNAs are highly complementary to specific miRNAs (such as CDR1as and miR-671), AGO2 can catalyze endonucleolytic slicing, directly resulting in circRNA breakage and clearance. In recent years, this mechanism has been revalidated in systems including neural tissues and cancer models, providing a unified explanation for the dual “sponge–slicing” outcomes: once the interaction shifts from “loose adsorption” to “near-perfect complementarity,” degradation becomes the predominant fate (Feng et al., 2023).

Another mechanism, termed structure-mediated RNA decay (SRD), represents a “shape-sensing” pathway independent of sequence features. SRD relies on UPF1 RNA helicase and G3BP stress granule assembly factor 1 (G3BP1) to recognize highly ordered or folded circRNA structures and promote endonucleolytic cleavage, thereby selectively reducing the steady-state levels of structurally complex circRNAs. Initially described at the transcriptome-wide level, subsequent studies in diverse model systems have suggested that interfering with UPF1 or G3BP1 preferentially upregulates highly structured circRNAs, indicating that SRD contributes to balancing the “structural diversity” of the circular RNAome (Fischer et al., 2020; Ren et al., 2022).

A major advance reported in 2025 was the identification of the ribonuclease DIS3, independent of exosomes, as a broad-spectrum circRNA degradation factor. Inhibition or depletion of DIS3 across multiple cell lines and animal tissues led to the upregulation of more than half of detected circRNAs, with minimal impact on their linear counterparts. This suggests that DIS3 exerts relatively “preferential” endonucleolytic activity toward circular forms. Further subcellular fractionation experiments supported its cytoplasmic function independent of exosomal pathways. These findings provide strong evidence that DIS3 represents a leading candidate for a “universal circRNA degradation pathway” under homeostatic conditions (Latini et al., 2025; Tao et al., 2025).

It is important to emphasize that these pathways do not operate in isolation. m6A modifications can alter the spatial conformation and binding repertoire of circRNAs, thereby influencing their routing between the YTHDF2–HRSP12–RNase P/MRP pathway and the SRD/UPF1–G3BP1 pathway. Innate immune activation, through RNase L, can induce global circRNA clearance that temporarily suppresses the fine-tuned regulation of circRNA pools by other homeostatic pathways. Meanwhile, AGO2/miRNA slicing reflects greater tissue- or developmental-stage specificity (Liu F. et al., 2024). Depending on tissue type, age, and pathological stress, the relative weighting and hierarchy of these regulatory axes can shift, shaping the heterogeneous “landscape of circRNA degradation” *in vivo* (Liu F. et al., 2024; Karimi et al., 2025).

## 3 Regulatory networks of circRNAs in diabetic cardiomyopathy

### 3.1 The regulatory mechanism of circRNAs in glucose metabolism disorders

In the pathogenesis of DCM, insulin resistance and chronic hyperglycemia collectively lead to significant disturbances in myocardial glucose metabolism (Mann et al., 2025). Under physiological conditions, cardiomyocytes rely primarily on the insulin–PI3K/Akt signaling pathway to mediate the translocation of glucose transporter type 4 (GLUT4) to the plasma membrane, thereby maintaining normal glucose uptake and energy homeostasis (Ferreira et al., 2018). However, under pathological DCM conditions, impaired insulin signaling disrupts GLUT4 translocation, markedly reducing glucose uptake efficiency in cardiomyocytes (Bertrand et al., 2020). Prolonged hyperglycemia also facilitates the aberrant accumulation of advanced glycation end-products (AGEs), which bind to their receptor (RAGE) and further suppress insulin signaling, forming a vicious cycle of “hyperglycemia–insulin resistance” (Giraldo-Gonzalez et al., 2025).

Recent studies have identified various circRNAs involved in the fine-tuned regulation of glucose metabolism, contributing to the pathological progression of DCM. For instance, circRNA_0071336 modulates glucose metabolic homeostasis in cardiomyocytes by sponging miR-93-5p and regulating GLUT4 expression (Yan et al., 2022). circIGF1R significantly influences cardiac fibroblast proliferation by modulating key enzymes associated with carbohydrate metabolism (Schmidt et al., 2025). Circ_0000284 specifically inhibits GLUT4 translocation in hepatocytes and contributes to arsenic-induced insulin resistance in a type 2 diabetes mellitus (T2DM) model (Xu S. et al., 2024). In another study, circHIPK3 promoted hyperglycemia and insulin resistance by sponging miR-192-5p and upregulating the transcription factor FOXO1, providing new insights and therapeutic targets for glucose metabolic disorders (Cai et al., 2020). Moreover, circPIP5K1A ameliorates insulin resistance, lipid metabolic disturbances, and inflammatory responses by targeting miR-552-3p to regulate ENO1 expression (Song G. et al., 2024). These findings advance understanding of circRNA-mediated regulation in glucose metabolism and support their potential as early diagnostic biomarkers or therapeutic targets in DCM.

### 3.2 circRNA regulation of cardiac hypertrophy

Cardiac hypertrophy is a common pathological change in DCM. Within this context, multiple studies report significant alterations in circRNA expression in cardiac tissue and circulation. For example, the conserved circRNA DICAR is downregulated in diabetic hearts; its deficiency leads to spontaneous cardiac dysfunction, cardiomyocyte hypertrophy, and fibrosis, whereas DICAR overexpression alleviates the DCM phenotype, suggesting a protective role in myocardial homeostasis (Yuan et al., 2023).

At the molecular level, circRNAs often function as competing endogenous RNAs (ceRNAs) or miRNA sponges, regulating pro- or anti-hypertrophic signaling. By adsorbing miRNAs that target pro-hypertrophic genes, circRNAs relieve suppression of these targets, promoting increased cell size and protein synthesis, ultimately driving cardiac hypertrophy (Cheng et al., 2024; Xu Z. et al., 2024).

CircRNAs also regulate programmed cell death and metabolic reprogramming under diabetic stress conditions (hyperglycemia, lipotoxicity, insulin resistance). Certain circRNAs influence mitochondrial function, oxidative stress, or inflammatory pathways, modulating cardiomyocyte susceptibility to apoptosis, pyroptosis, or PANoptosis. For instance, circ-OGDH can promote or regulate PANoptosis in cardiomyocytes, indirectly influencing hypertrophy and functional impairment (Guan et al., 2025).

Myocardial interstitial remodeling and fibroblast activation, closely associated with hypertrophy, are also regulated by circRNAs. By altering fibroblast metabolism or functioning within ceRNA networks, circRNAs modulate collagen synthesis and fibroblast proliferation. Targeting pro-fibrotic circRNAs attenuates fibroblast activation and ventricular wall thickening, highlighting circRNAs as key regulators of fibrotic signaling and hypertrophic progression (You et al., 2025). Some circRNAs additionally act *via* noncanonical mechanisms, interacting with myosin-associated proteins to regulate protein stability or translation. For example, circ-0001283 exacerbates cardiac hypertrophy by modulating myosin light chain, illustrating mechanistic diversity and context-dependence (Wang W. et al., 2025).

The impact of circRNAs on hypertrophic phenotypes is highly dependent on tissue type, disease stage, and pathological stressors. Biphasic or opposing effects may occur for the same circRNA in different models or disease phases. High-throughput sequencing and functional screens have identified numerous circRNAs related to metabolism, cell death, and fibrosis, but challenges such as specificity, delivery, safety, and cross-species conservation remain before clinical translation (Mei et al., 2024; Yao et al., 2024).

### 3.3 The role of circRNAs in oxidative stress and inflammatory responses

Oxidative stress and subsequent inflammation are hallmark pathological features of DCM, primarily initiated by excessive reactive oxygen species (ROS) under hyperglycemia (Chen and Guo, 2025). Mitochondrial electron transport chain (ETC.) dysfunction from imbalanced glucose and fatty acid oxidation leads to electron leakage, generating superoxide (O_2_•^-^) (Li T. et al., 2025). Hyperglycemia also activates NADPH oxidase (NOX), especially NOX2/NOX4, while AGEs binding to RAGE amplifies ROS production *via* a positive feedback loop (Li T. et al., 2025). Excess ROS oxidatively modifies IKKβ kinase, triggering IκBα degradation and NF-κB nuclear translocation, inducing transcription of pro-inflammatory cytokines such as TNF-α and IL-6. Activation of the NLRP3 inflammasome *via* caspase-1 promotes IL-1β and IL-18 maturation, directly damaging cardiomyocytes and activating fibroblasts, leading to ECM deposition and fibrosis (Peng et al., 2022). ROS-induced lipid peroxidation, protein carbonylation, and DNA damage ultimately impair cardiomyocyte structure and function, resulting in systolic and diastolic dysfunction (Lei et al., 2025).

CircRNAs participate in regulating oxidative stress in DCM through multiple mechanisms. CircHIPK3 promotes ROS generation by sponging miR-20b-5p and upregulating ATG7 (Qiu et al., 2021). CircSlc8a1 enhances oxidative stress in H9c2 cells *via* the miR-673-5p/TFRC axis (Wu and Du, 2024). Circ-AMOTL1 is upregulated in diabetic myocardium, and its silencing improves cardiac function, reduces fibrosis, and decreases MARCKS expression, indicating pathogenic involvement (Yang Y. et al., 2023). Conversely, circFOXP1 exerts cardioprotective effects by lowering ROS through miR-9-3p binding (Rong et al., 2025).

CircRNAs also modulate inflammatory responses in DCM. CircPIP5K1A upregulation in STZ-induced diabetic rats correlates with inflammation, and its downregulation alleviates insulin resistance and inflammation *via* miR-552-3p/ENO1 regulation (Song G. et al., 2024). Circ_0003928 regulates high-glucose-induced oxidative stress and inflammation *via* the miR-31-5p/MAPK6 axis in HK-2 cells (Bao et al., 2024). CircANKRD36 silencing in T2DM rats upregulates miR-145, targets XBP1, and mitigates inflammation (Lu et al., 2021). Studies in diabetic nephropathy also highlight circRNA involvement in inflammation and fibrosis, e.g., circ-ITCH *via* miR-33a-5p/SIRT6 and circTAOK1 *via* miR-142-3p/SOX6 axes (Liu et al., 2021; Liu SY. et al., 2024). These renal findings provide mechanistic insights for cardiac circRNA research and therapeutic development in DCM.

### 3.4 The role of circRNAs in myocardial fibrosis

Myocardial fibrosis is a critical pathological feature of DCM, characterized by excessive ECM accumulation, particularly collagen. Chronic hyperglycemia and metabolic dysregulation activate cardiac fibroblasts, enhancing ECM synthesis and impairing degradation, resulting in structural and functional myocardial impairment (Sun J. et al., 2025). AGEs binding to RAGE activate pro-fibrotic pathways, including TGF-β/Smad signaling, while ROS further enhance fibrosis (Qin et al., 2025; Yue et al., 2017). Insulin resistance disrupts energy and lipid metabolism, promoting apoptosis and reparative fibrosis (Xu H. et al., 2024).

Fibrotic remodeling increases myocardial stiffness and reduces ventricular compliance, causing diastolic dysfunction (Smati et al., 2025). Progressive fibrosis replaces cardiomyocytes, reducing contractile units and resulting in systolic dysfunction and heart failure. Fibrosis also disrupts electrical conduction, increasing arrhythmia risk (Cunha et al., 2022).

CircHIPK3 is widely expressed in the heart, liver, and brain and is upregulated in DCM, promoting myocardial fibrosis *via* miR-152-3p/TGF-β2 and miR-29b-3p pathways (Wang et al., 2021; Liu et al., 2020). High-throughput sequencing identified circPHF20L1, circCLASP1, and circSLC8A1 as key regulators, with axes including circCLASP1/miR-182-5p/Wnt7a, circSLC8A1/miR-29b-1-5p/Col12a1, and circPHF20L1/miR-29a-3p/Col6a2 (Yuan et al., 2024). Circ-AMOTL1 and circRNA_010567 also contribute to fibrosis *via* MARCKS and TGF-β1 regulation, respectively (Yuan et al., 2024; Zhou and Yu, 2017).

**FIGURE 2:**
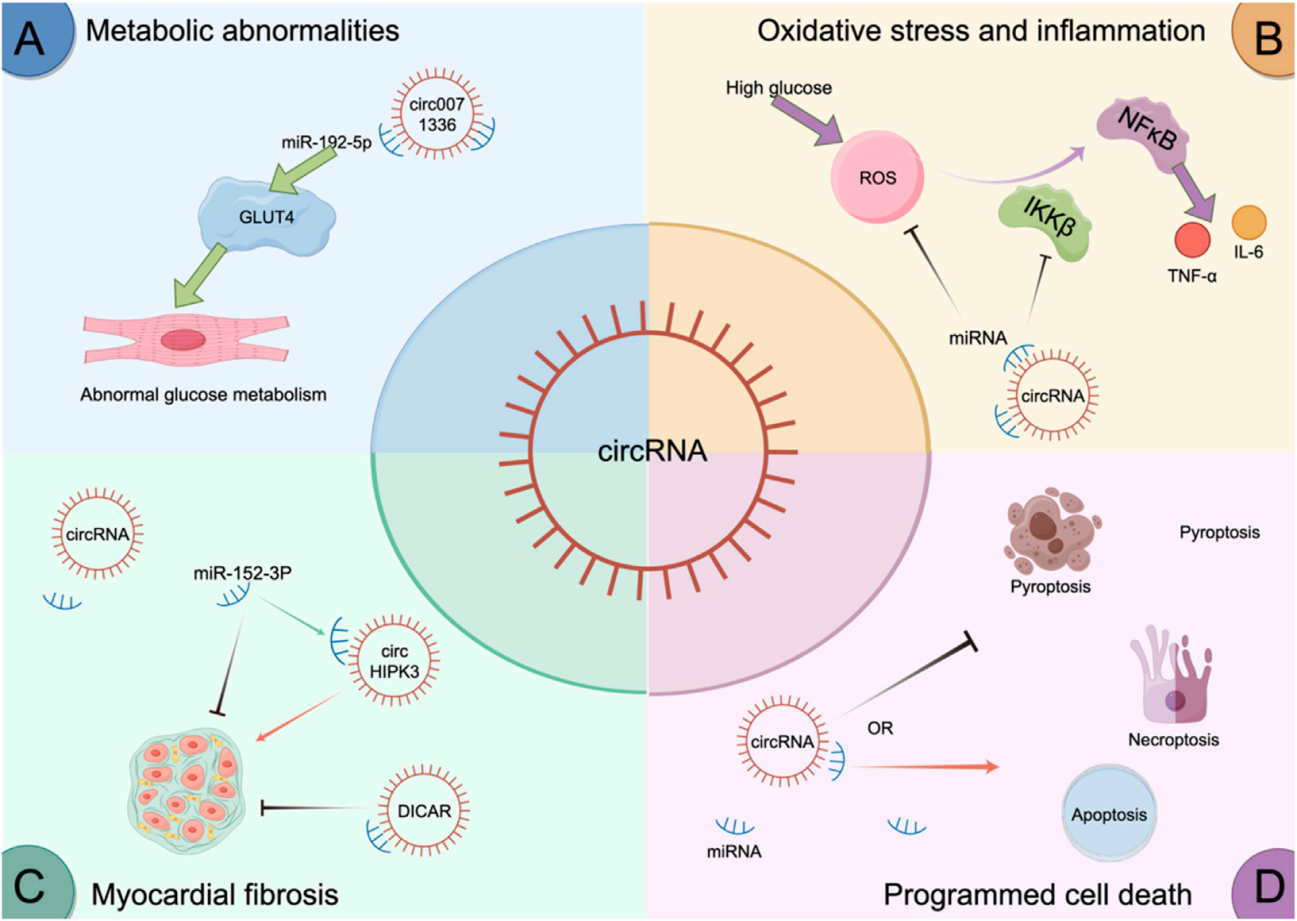
circRNAs regulate DCM.

Some circRNAs exert anti-fibrotic effects. DICAR overexpression alleviates fibrosis through a VCP-Med12–mediated degradation pathway (YUAN et al., 2023). CircRNA_012164 and circRNA_42623 regulate fibrosis *via* miR-9-5p–related pathways, and knockdown reverses fibrotic phenotypes (WANG H. et al., 2024; WANG H. et al., 2022).The mechanism by which circRNAs regulate DCM is shown in Figure 2.

### 3.5 The role of circRNA in cardiomyocyte death

Programmed cell death (PCD) of cardiomyocytes plays a central role in the onset and progression of DCM (Zhang et al., 2024). High-glucose-induced PCD contributes not only to cardiomyocyte loss but also aggravates inflammation and promotes myocardial fibrosis, collectively accelerating cardiac dysfunction (Xuan and Zhang, 2023). PCD encompasses various cell death modalities, including apoptosis, autophagy, pyroptosis, and necroptosis, which interact to form a complex regulatory network in DCM pathogenesis.

Emerging evidence suggests that high-glucose conditions alter the expression profiles of numerous circRNAs, which modulate specific PCD pathways affecting cardiomyocyte viability and function. For instance, CDR1as, a circRNA with prognostic potential, is upregulated in DCM, while its knockdown significantly attenuates cardiomyocyte apoptosis. Mechanistic studies reveal that CDR1as inhibits ubiquitination of mammalian sterile 20-like kinase 1 (MST1), thereby activating the Hippo signaling pathway to suppress apoptosis (Shao et al., 2022). Similarly, circMAP3K5 is upregulated in response to hyperglycemia and promotes apoptosis of H9c2 cells by sponging miR-22-3p and upregulating death-associated protein kinase 2 (DAPK2), identifying the circMAP3K5/miR-22-3p/DAPK2 axis as a potential therapeutic target (Shen et al., 2024). CircHIPK3 also plays an anti-apoptotic role by downregulating PTEN, a negative regulator of survival pathways, thereby protecting AC16 cells from high-glucose-induced apoptosis (Jiang et al., 2022). High-throughput RNA sequencing has further identified several apoptosis-related circRNAs—such as mmu_circ_0000652, mmu_circ_0000547, mmu_circ_0001058, mmu_circ_0000680, and novel_circ_0004285—that may modulate early-stage diabetic myocardial apoptosis through competitive miRNA binding (Dong et al., 2020).

In terms of pyroptosis, circ_0071269 is significantly upregulated in H9c2 cells under high-glucose conditions (Fu et al., 2022). It promotes pyroptosis and inflammation *via* the miR-145/GSDMA axis, while its knockdown mitigates cytotoxicity and enhances cell viability (Fu et al., 2022). DICAR, previously described as a protective circRNA in DCM, inhibits cardiomyocyte pyroptosis, potentially *via* a VCP-Med12 degradation mechanism (Yuan et al., 2023). Another pyroptosis-associated circRNA, CACR (hsa_circ_0076631), is elevated in both diabetic patient serum and high-glucose-treated cardiomyocytes (Yang et al., 2019). CACR sponges miR-214-3p, relieving suppression of caspase-1, thereby enhancing pyroptotic signaling. CACR knockdown attenuates caspase-1 activation, while miR-214-3p inhibition partially reverses this effect, supporting the therapeutic relevance of the CACR/miR-214-3p/caspase-1 axis (Yang et al., 2019). PYRCR, another pyroptosis-related circRNA, protects against ischemia/reperfusion (I/R)-induced cardiac injury by modulating Drp1 activity *via* DRG2, offering a novel strategy for pyroptosis inhibition (Chen X. Z. et al., 2025).

**FIGURE 3:**
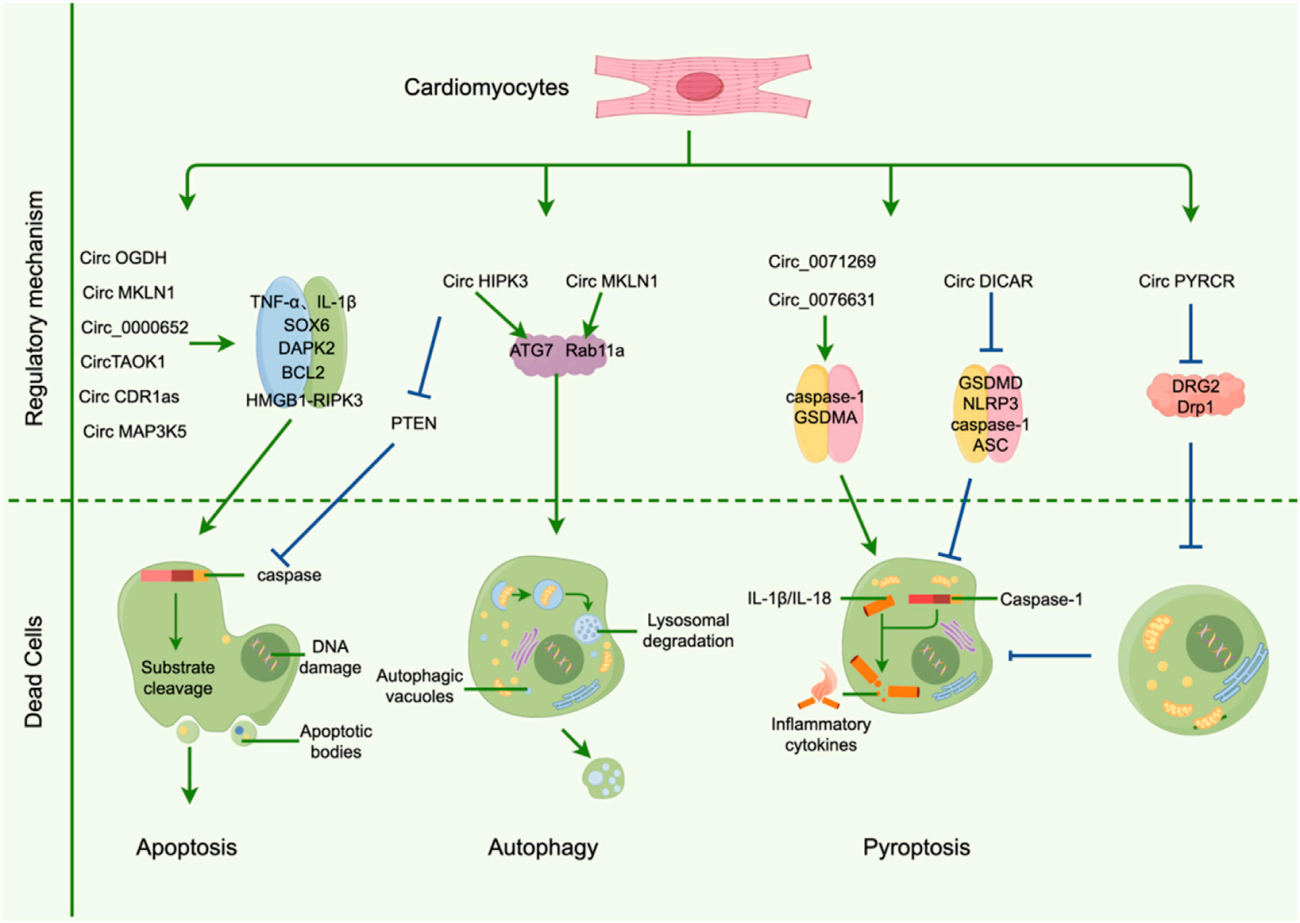
CircRNAs regulate the mechanism of programmed cardiomyocyte death.

In other PCD types, circOGDH expression is elevated in diabetic mouse myocardium, accompanied by upregulation of PANoptosis-related proteins (Guan et al., 2025). CircOGDH specifically regulates RIPK3 *via* the HMGB1 pathway, triggering necroptosis and exacerbating cardiac injury, providing mechanistic insights into DCM (Guan et al., 2025). Regarding autophagy, circMKLN1 is upregulated in serum of STZ-induced diabetic mice and acts as a sponge for miR-26a-5p to regulate autophagy in high-glucose/methylglyoxal-treated human retinal microvascular endothelial cells (hRMECs) (Yang J. et al., 2023). Silencing circMKLN1 inhibits excessive autophagy and inflammation, offering novel clues for exploring aberrant autophagy in cardiomyocytes and its role in DCM progression (YANG J. et al., 2023). The mechanism of CircRNAsPCD is shown in Figure 3.

### 3.6 Bidirectional regulation of circRNA in DCM

It is noteworthy that in the pathological progression of DCM, different circRNAs may exert diametrically opposed effects. Several studies have demonstrated that under comparable conditions of hyperglycemia, hypoxia, or oxidative stress, some circRNAs, such as circFOXP1, exhibit cardioprotective effects by suppressing oxidative stress, attenuating apoptosis, and mitigating inflammatory responses, whereas others, such as circHIPK3, may aggravate disease progression by inducing mitochondrial fission, increasing ROS production, and directly damaging cardiomyocytes. These functional discrepancies may result from the interplay of several molecular mechanisms.

First, subcellular localization and target site specificity are critical determinants of circRNA functional diversity. Certain circRNAs localize to mitochondria or interact with proteins regulating mitochondrial dynamics—for instance, circHIPK3 promotes mitochondrial fission and elevates ROS levels by acting on DRP1—thereby directly amplifying oxidative stress (Li X. et al., 2025). In contrast, other circRNAs reside in the cytoplasm or nucleus and function through sponging miRNAs or interacting with RNA-binding proteins (RBPs), suppressing pro-oxidative or pro-apoptotic pathways and thus conferring protective effects (Rong et al., 2025).

Second, the functional output of circRNAs is strongly influenced by the abundance and binding affinity of miRNAs within the competing endogenous RNA (ceRNA) network. Whether a circRNA can effectively “release” its downstream targets depends on the number of miRNA binding sites, the binding affinity, and the cellular expression level of the circRNA itself. In contexts where miRNAs are highly expressed or target gene networks are complex, the sponging capacity of certain circRNAs may be markedly diminished, leading to a weak or absent protective phenotype.

Third, RBP- and splicing factor–mediated functional switching is also crucial. CircRNAs may serve as scaffolds or molecular decoys for RBPs, or indirectly regulate their host linear transcripts or other genes by altering RBP accessibility. Under different pathological conditions, alterations in the expression or activity of splicing factors such as the serine/arginine-rich splicing factor (SRSF) family or polypyrimidine tract-binding protein 1 (PTBP1) may further influence circRNA biogenesis, isoform composition, and functional orientation. For example, SRSF4 has been reported to associate with cardiac circFOXP1 and modulate its downstream functions (Song et al., 2025). Thus, the same circRNA may display protective or pathogenic phenotypes depending on the splicing or binding context.

In addition, epitranscriptomic modifications such as N^6^-methyladenosine (m6A) and translational potential profoundly shape circRNA functions. m6A modification not only regulates circRNA stability and nucleo-cytoplasmic distribution but also facilitates cap-independent translation, resulting in functional peptides or altered RBP-binding profiles, thereby broadening circRNA-mediated effects. Under pathological conditions, altered expression of m^6A-associated enzymes, including methyltransferase-like proteins (METTL3/14), demethylases (FTO/ALKBH5), and YTH domain-containing family (YTHDF) reader proteins, may dynamically reprogram the functional properties of specific circRNAs, shifting them between roles as “noncoding RNAs” and “peptide-coding transcripts,” and ultimately producing divergent phenotypes (Qin et al., 2022).

Moreover, disease stage and expression kinetics cannot be overlooked. CircRNAs that are upregulated in the early stress response may serve compensatory protective roles, such as transiently suppressing apoptosis or promoting repair; however, sustained overexpression in a chronic hyperglycemic environment may convert them into pathogenic drivers that exacerbate inflammation or fibrosis. This time- and dose-dependent effect explains why cross-sectional studies often yield inconsistent results and underscores the necessity of longitudinal and functional studies.

Finally, methodological variability may amplify apparent contradictions across studies. Model selection (e.g., streptozotocin [STZ]-induced mice, high-fat diet [HFD] models, *in vitro* high-glucose or oxidative stress treatment), endpoints of observation (transcriptional levels, ROS detection, apoptosis rate, or cardiac function), and intervention strategies (knockdown, overexpression, antisense oligonucleotides [ASO], or CRISPR-Cas13 approaches) all influence the comparability of findings (Yao et al., 2024; Qin et al., 2022). The research progress of circular RNAs is shown in Table 1.

**TABLE 1:** Summary of Functional Studies on circRNAs in DCM.

circRNA name	Research species or cell type	Downstream target gene/Pathway	Functional phenotype	References
circ_0071336	Human	GLUT4	Targets miRNA miR-93-5p, maintains glucose metabolic homeostasis	Yan et al. (2022)
circIGF1R	HCFs	AZGP1	Directly targets fibroblast metabolism to alleviate cardiac fibrosis	Schmidt et al. (2025)
circ_0000284	HepG2	IGF2BP2/PPAR-γ	Blocks plasma membrane translocation of GLUT4 in hepatocytes, involved in arsenic-induced hepatic insulin resistance	Xu et al. (2024b)
circHIPK3	Rat, Mouse, HepG2, Huh7, CFs	FOXO1, ATG7, Col1a1, Col3a1, TGF-β2, PTEN	Sponges miR-192-5p, promoting hyperglycemia and insulin resistance; accelerates cardiomyocyte autophagy and apoptosis during myocardial I/R injury; targets miR-29b-3p and miR-152-3p to promote cardiac fibrosis; downregulates PTEN to regulate cardiomyocyte apoptosis	Cai et al. (2020), Wang et al. (2021), Liu et al. (2020), Jiang et al. (2022)
circPIP5K1A	Rat	ENO1	Targets miR-552-3p to mediate ENO1 expression, ameliorates insulin resistance and lipid metabolism disorders; inhibits inflammation	Song et al. (2024b), Qiu et al. (2021)
circSlc8a1	H9c2, Mouse	TFRC, Col12a1	Enhances oxidative stress in cardiomyocytes; targets miR-29b-1-5p to regulate cardiac fibrosis	Wu and Du (2024)
circAMOTL1	Mouse, CFs	MARCKS	Promotes myocardial interstitial and perivascular fibrosis; promotes cell proliferation, fibrotic marker proteins, and levels of ROS and NO	Yang et al. (2023a)
circFOXP1	Human, Mouse	TNF-α, IL-1β, IL-6	Targets miR-9-3p to inhibit apoptosis, inflammation, and oxidative stress	Rong et al. (2025)
circ_0003928	HK-2	MAPK6	Targets miR-31-5p to promote high glucose-induced inflammation and oxidative stress	Bao et al. (2024)
circANKRD36	Rat	XBP1	Alleviates insulin resistance and inflammation	Lu et al. (2021)
circITCH	Mouse	SIRT6	Targets miR-33a-5p to alleviate renal inflammation and fibrosis in STZ-induced diabetic mice	Liu et al. (2021)
circTAOK1	HK-2	SOX6	Targets miR-142-3p to regulate high glucose-induced inflammation, oxidative stress, ECM accumulation, and apoptosis in diabetic nephropathy	Liu et al. (2024b)
circCLASP1	Mouse	Wnt7a	Targets miR-182-5p to regulate cardiac fibrosis	Yuan et al. (2024)
circPHF20L1	Mouse	Col6a2	Targets miR-29a-3p to regulate cardiac fibrosis	Yuan et al. (2024)
circ_010567	Mouse	TGF-β1	Targets miR-141 to promote cardiac fibrosis	Zhou and Yu (2017)
circDICAR	H9c2, Mouse	GSDMD, NLRP3, caspase-1, ASC	Inhibits diabetic cardiomyocyte pyroptosis and cardiac fibrosis	Wang et al. (2024b)
circ_012164	Mouse, MCECs	miR-9	Promotes cardiac fibrosis	Wang et al. (2022c)
circCDR1as	Mouse	MST1, Hippo pathway	Promotes apoptosis induced by DCM	Shao et al. (2022)
circMAP3K5	Rat	DAPK2	Targets the miR-22-3p/DAPK2 axis to promote high glucose-induced cardiomyocyte apoptosis	Shen et al. (2024)
circ_0000652	Mouse	BCL2	Targets miR-195 to regulate cardiomyocyte apoptosis	Dong et al. (2020)
circ_0001058	Mouse	SPRY1	Targets miR-21 to regulate myocardial interstitial fibrosis	Dong et al. (2020)
circ_0001160	Mouse	ZNT7	Encodes a protein that intervenes in early DCM progression	Dong et al. (2020)
circ_0071269	H9c2, Mouse	GSDMA	Promotes myocardial pyroptosis in DCM	Fu et al. (2022)
circ_0076631	Mouse	caspase-1	Promotes myocardial pyroptosis in DCM	Yang et al. (2019)
circPYRCR	Mouse	DRG2, Drp1	Inhibits mitochondrial fission, cardiomyocyte pyroptosis, and myocardial injury	Chen et al. (2025b)
circOGDH	Mouse	HMGB1-RIPK3 signaling pathway	Promotes apoptosis and inhibits PANoptosis	Guan et al. (2025)
circMKLN1	Mouse	Rab11a	Promotes reactive autophagy and inflammation	Yang et al. (2023b)

Abbreviations: HCFs, Human Cardiac Fibroblasts; CFs, Cardiac Fibroblasts; MCECs, Mouse Cardiac Endothelial Cells; STZ, Streptozotocin; I/R, Ischemia/Reperfusion; ECM, Extracellular Matrix; DCM, Diabetic Cardiomyopathy.

## 4 Prospects and challenges of clinical translation of circRNAs

In recent years, the expanding body of research on circRNAs in DCM has provided novel insights into the pathogenesis of this complex metabolic cardiac disorder. Owing to their unique closed-loop structure, circRNAs exhibit high stability, tissue specificity, and evolutionary conservation, highlighting their promising potential for clinical applications. From a translational perspective, the utility of circRNAs in DCM is primarily reflected in three aspects: as non-invasive biomarkers, as therapeutic targets, and as tools for disease stratification and individualized treatment.

Firstly, circRNAs exhibit distinct expression alterations in both myocardial tissues and peripheral blood under diabetic conditions, suggesting their potential as non-invasive diagnostic and prognostic biomarkers (Dong et al., 2020; Imran et al., 2024). For example, circRNAs such as CACR, CDR1as, and circHIPK3 demonstrate specific expression patterns in both animal models and patient sera, supporting their application in early detection and disease monitoring of DCM (Wang et al., 2021; Shao et al., 2022; Yang et al., 2019; Shao et al., 2024). Secondly, circRNAs can regulate downstream gene expression, making them attractive candidates for novel therapeutic targets. In the future, synthetic circRNA mimics or inhibitory molecules (e.g., siRNAs or ASOs) may be utilized to modulate circRNA function and thereby intervene in pathological processes (Maziec et al., 2025). However, clinical implementation of circRNA-based therapies remains challenging. Issues such as efficient and targeted delivery systems, off-target effects, and immunogenicity require further investigation. Moreover, circRNA expression profiling could facilitate DCM patient stratification and precision therapy (Li et al., 2024; Li S. Y. et al., 2025). In-depth characterization of circRNA-mediated molecular networks may help identify subgroups of diabetic patients who are more susceptible to myocardial injury or more responsive to specific therapeutic strategies, thus promoting the integration of precision medicine into cardiovascular-metabolic disease management (Zhao R. et al., 2025).

## 5 Limitations and perspectives

### 5.1 Current methodological challenges in studying circRNAs in DCM

Although significant progress has been made in elucidating the role of circRNAs in DCM—particularly in regulating programmed cardiomyocyte death, inflammatory responses, mitochondrial dysfunction, and fibrosis—the field remains in its infancy and faces several unresolved challenges that demand further investigation. Current studies exploring the relationship between circRNAs and DCM predominantly rely on *in vitro* stress models and animal systems, yet these approaches have inherent methodological constraints.

First, precise quantification of circRNAs in cardiac tissue remains challenging. Conventional sequencing combined with RNase R enrichment is prone to bias, short-read sequencing cannot discriminate between isoforms, and single-cell or spatial approaches are limited by sequencing depth, all of which compromise the accuracy of expression profiling (Nielsen et al., 2022; Vromman et al., 2025). Second, validation of targets and mechanisms is insufficient. Most studies rely on predictive models of “miRNA sponging” or RBP interactions without robust supporting evidence from CLIP assays, reporter systems, or stoichiometric analyses, leaving their *bona fide* roles in the myocardium uncertain. Cell-type heterogeneity and dynamic changes in splicing factors further complicate interpretation (MontAñés-Agudo et al., 2023). Third, *in vivo* functional studies remain limited. While siRNA or ASOs are widely used, they often fail to distinguish circular from linear transcripts. Newer approaches such as circular-specific ASOs (cASOs) and CRISPR-Cas13 have improved specificity, yet challenges remain in delivery efficiency, off-target effects, and immunogenicity (Ai et al., 2022; Shi and Wu, 2024). Finally, the choice of models and endpoints significantly influences extrapolation. STZ, db/db, and high-fat diet (HFD) models differ in their metabolic and inflammatory features, while *in vitro* hyperglycemia or ROS stimulation poorly recapitulates the complex cardiac milieu. Moreover, many studies assess transcript levels or oxidative stress indices without direct correlation to cardiac structure or function. Collectively, these factors contribute to inconsistent findings across studies, underscoring the need for integration of long-read and single-cell sequencing, targeted interventions, and longitudinal functional evaluation to enhance both reliability and translational relevance (Bibi et al., 2025).

### 5.2 Limitations of cross-model extrapolation and the need for cardiac-specific validation

Given the current paucity of circRNA-focused studies in DCM, this review has, in part, referenced findings from other disease models such as diabetic kidney disease (DKD) and myocardial ischemic injury. These conditions share common pathological features with DCM, including chronic hyperglycemia and oxidative stress, which drive mitochondrial dysfunction and excessive ROS production, ultimately triggering apoptosis, autophagy, and inflammatory responses. Moreover, they all involve dysregulation of noncoding RNA networks, including circRNAs, contributing to fibrosis, cell death, and metabolic derangements. These shared mechanisms provide a rationale for cross-model comparison, offering useful insights into the potential roles of circRNAs in DCM.

Nevertheless, caution is warranted when extrapolating from external models to DCM. Pathological contexts differ significantly, shaping both circRNA expression and function. In DKD, the major drivers include glomerular hyperglycemic stress and basement membrane remodeling, whereas ischemia/reperfusion (I/R) injury reflects acute hypoxia-reoxygenation. In contrast, DCM is characterized by chronic hyperglycemia, insulin resistance, metabolic reprogramming, and AGE accumulation. These differences mean that the same circRNA may exhibit distinct phenotypes across tissues or models, depending on variations in cell composition, miRNA/RBP backgrounds, and metabolic state (Liu and Zhao, 2022; Pan M. et al., 2025). DCM also exhibits unique features that limit direct extrapolation, including cardiomyocytes’ high dependency on energy metabolism and calcium homeostasis, the central role of mitochondrial dysfunction, and a prolonged course of metabolic inflammation and fibrosis. These factors may reshape circRNA biology—affecting subcellular localization, miRNA sponging capacity, and RBP interactions—ultimately altering whether their effects in the myocardium are pathogenic or protective (Fang et al., 2025).

To improve extrapolative validity, validation in cardiac-specific contexts is essential. This includes reproducing circRNA expression profiles and functions in myocardial tissue or isolated cardiac cell types (cardiomyocytes, fibroblasts, endothelial, or immune cells); applying single-cell/spatial transcriptomics and long-read sequencing to resolve isoforms and subcellular localization; and employing back-splice junction (BSJ)-specific interventions (e.g., cASOs or optimized CRISPR-Cas13 platforms) delivered *via* AAV9 or other cardiotropic systems, with rigorous evaluation of dose–response, off-target activity, and immunogenicity (Guan et al., 2025; Wu et al., 2023).

### 5.3 Challenges and future directions towards clinical application

In the future, promoting the clinical application of circRNAs in DCM will require overcoming critical challenges in both human sample research and therapeutic delivery. The procurement of human cardiac tissue is constrained by ethical considerations and limited availability, while serum circRNA levels are susceptible to individual variability and comorbidities, resulting in inconsistent detection reliability (Yan et al., 2024). Therefore, relying solely on differential expression profiles is insufficient to establish causality. Integrating single-cell omics, spatial transcriptomics, and CRISPR-based functional validation technologies will be essential to elucidate the precise role of circRNAs in disease mechanisms.

Simultaneously, the complex cardiac microenvironment imposes stringent demands on therapeutic delivery systems. Current delivery vehicles are generally limited by issues such as immunogenicity, low efficiency, and lack of cell-type specificity. Future efforts should prioritize developing nano-delivery systems with low immunogenicity, controllable release kinetics, and cardiomyocyte-specific targeting to enable efficient and clinically safe translation of circRNA-based therapies.

In summary, circRNAs retain significant promise as both potential biomarkers and therapeutic targets for DCM, underscoring their continued value in further research and clinical translation.

## Glossary

DCM: Diabetic cardiomyopathy
circRNAs: circular RNAs
GLUT4: glucose transporter type 4
AGEs: advanced glycation end-products
RAGE: receptor for advanced glycation end-products
miRNA: microRNA
ceRNA: competing endogenous RNA
ROS: reactive oxygen species
NOX: NADPH oxidase
ECM: extracellular matrix
STZ: streptozotocin
HFD: high-fat diet
ASOs: antisense oligonucleotides
cASOs: circular-specific antisense oligonucleotides
BSJ: back-splice junction
PCD: programmed cell death
ASK1: apoptosis signal-regulating kinase 1
DAPK2: death-associated protein kinase 2
DRP1: mitochondria fission regulator
hRMECs: human retinal microvascular endothelial cells
METTL3/14: methyltransferase-like proteins
FTO: fat mass and obesity-associated protein
ALKBH5: AlkB homolog 5
YTHDF: YTH domain family proteins
I/R: ischemia/reperfusion
DKD: diabetic kidney disease
MST1: mammalian sterile 20-like kinase 1
Hippo: Hippo signaling pathway
MARCKS: myristoylated alanine-rich C kinase substrate
STZ-T1DM: streptozotocin-induced type 1 diabetes mellitus
T2DM: type 2 diabetes mellitus
PTBP1: polypyrimidine tract-binding protein 1
SRSF: serine/arginine-rich splicing factor
IR: insulin resistance
TF: transcription factor
HG: high-glucose
Akt: protein kinase B
PI3K: phosphoinositide 3-kinase
TGF-β/Smad: TGF-β/Smad signaling pathway
LPO: reactive oxygen species-induced lipid peroxidation
NF-κB: nuclear factor kappa B
NLRP3: NLR family pyrin domain containing 3
